# Development of Multiplex RT qPCR Assays for Simultaneous Detection and Quantification of Faecal Indicator Bacteria in Bathing Recreational Waters

**DOI:** 10.3390/microorganisms12061223

**Published:** 2024-06-18

**Authors:** Marina Carrasco-Acosta, Pilar Garcia-Jimenez

**Affiliations:** Department of Biology, Faculty of Marine Sciences, Instituto Universitario de Investigación en Estudios Ambientales y Recursos Naturales i-UNAT, Universidad de Las Palmas de Gran Canaria, 35017 Las Palmas de Gran Canaria, Spain; marina.carrasco@ulpgc.es

**Keywords:** *Escherichia coli*, intestinal enterococci, FIB, marine sediments, multiplex RT qPCR, 16S rRNA, *ybbW*

## Abstract

In this study, we designed and validated in silico and experimentally a rapid, sensitive, and specific multiplex RT qPCR for the detection and quantification of faecal indicator bacteria (FIB) used as microbiological references in marine bathing water regulations (*Escherichia coli* and intestinal enterococci). The 16S rRNA gene was used to quantify group-specific enterococci and *Escherichia/Shigella* and species-specific such as *Enterococcus faecalis* and *E. faecium*. Additionally, a *ybbW* gene encoding allantoin transporter protein was used to detect *E. coli*. An assessment of marine coastal systems (i.e., marine water and sediment) revealed that intestinal enterococci were the predominant group compared to *Escherichia*/*Shigella*. The low contribution of *E. faecalis* to the intestinal enterococci group was reported. As *E. faecalis* and *E. faecium* were reported at low concentrations, it is assumed that other enterococci of faecal origin are contributing to the high gene copy number of this group-specific enterococci. Moreover, low 16S rRNA gene copy numbers with respect to *E. faecalis* and *E. faecium* were reported in seawater compared to marine sediment. We conclude that marine sediments can affect the quantification of FIBs included in bathing water regulations. Valuing the quality of the marine coastal system through sediment monitoring is recommended.

## 1. Introduction

Seawater is vulnerable to faecal contamination as continuous population growth, climate change, and inadequate coastal management contribute to increased pollution. Moreover, the high incidence of water-related diseases [1,2,3,4], such as gastrointestinal and respiratory diseases [5,6,7,8] or skin disorders [9,10], renders marine waters globally relevant, as waterborne diseases can spread [11]. Therefore, the protection of coastal areas and their sanitary quality is an increasingly important global concern, as reflected in the 2030 Agenda, particularly the 14th goal—Life under Water—and, more recently, the Ocean Decade 2021–2030 [12,13]. Consequently, enhancing coastal monitoring and standards for assessing safety regulations would improve environmental and public health and also open up new socio-economic opportunities [14].

The referenced analysis method for the Bathing Water Directive (BWD; European Union 2006/7/CE) [15] is based on culture and counting faecal indicator bacteria (FIB), such as *Escherichia coli* and intestinal enterococci. These analyses based on culture-dependent methods require long diagnostic times (24–48 h), which implies a delay in decision-making when faecal contamination appears in recreational waters. In response, culture-independent methods based on DNA, such as simultaneous real-time quantitative PCR (multiplex RT qPCR) or conventional real-time quantitative PCR (RT qPCR), have been reported as promising methods because they allow the specific identification and quantification of microorganisms within a few hours [16,17,18], such as proposed RT qPCR assays by the U.S. Environmental Protection Agency for FIB detection [19]. Additionally, multiplex RT qPCR reduces the required sample volume, cost, and handling of assays compared to RT qPCR or other independent-culture methods [20,21,22].

Most nucleic acid-based approaches target the 16S rRNA gene with respect to the detection of FIBs [23,24,25,26], reporting relevant information at the group level for the Enterococcaceae family [20,23]. Nonetheless, other genes or target regions such as beta-glucuronidase [20,27] and allantoin permease [28,29,30,31] are also proposed as 16S rRNA sequences do not distinguish between close species belonging to the Enterobacteriaceae family, such as *Shigella* spp. and *E. coli*, which share phenotypic traits with separate entities relative to epidemiology and clinical disease. In contrast, the problem with enterococci is that they are a heterogeneous group, including faecal species, such as *Enterococcus faecalis* and *Enterococcus faecium*, and non-faecal species. *Enterococcus faecalis* and *E. faecium* have been extensively described as representatives of the genus *Enterococcus* in the human gastrointestinal tract [32,33,34,35], and they are also responsible for the majority of enterococcal infections in humans—*E. faecalis* at approximately 60% and *E. faecium* at approximately 40% [36,37]. 

Furthermore, coastal marine sediments are also influenced by the inputs of adjacent marine water and freshwater. Proximity to coastal habitats often increases organic matter for microbial growth, and sediments favour habitats for the growth of faecal bacteria [38,39]. Notwithstanding, marine sediments are not included in the European Bathing Water Directive [15].

The aim of this paper is to detect and quantify, through multiplex RT qPCR assays, faecal indicator bacteria, such as intestinal enterococci and *E. coli*, as microbiological references in marine bathing water regulations. These assays are performed via the in silico validation of primers and TaqMan probes through *16S rRNA* based on group-specific enterococci and *Escherichia/Shigella* and species-specific *Enterococcus faecalis* and *E. faecium*. Additionally, the *ybbW* gene encoding allantoin transporter protein is used to detect *E. coli*. Moreover, an evaluation of the quality of the marine coastal system through seawater and sediment monitoring is carried out. 

## 2. Materials and Methods

### 2.1. Target Selection and Design of Primers and TaqMan Probes 

*Escherichia coli* and intestinal enterococci were selected as the faecal indicators of recreational marine water quality according to BWD [15]. Assessments of FIBs were carried out to determine and quantify (i) the group-specific FIBs of *Escherichia*/*Shigella* and the main enterococci species of faecal origin with clinical interest (hereafter enterococci) and (ii) species-specific FIBs, such as *E. coli*, *Enterococcus faecalis*, and *Enterococcus faecium*. 

The 16S rRNA multicopy gene was selected to identify the group-specific FIBs of *Escherichia*/*Shigella*, intestinal enterococci, and the species *E. faecalis* and *E. faecium*. *E. coli* was revealed using a single-copy gene (*ybbW* gene)-encoding allantoin permease protein.

The allantoin gene provides 100% inclusivity and exclusivity identification within *E. coli* [28,29,30,31]. Primers and probes were designed from eleven 16S rRNA complete gene sequences of *Escherichia* spp. and *Shigella* spp. (Table S1) and fourteen 16S rRNA gene sequences of *Enterococcus* spp. of faecal origin and clinical importance (Table S2). Primers and probes for species-specific FIBs were designed from 68 complete 16S rRNA gene sequences for the *Enterococcus* species retrieved from the List of Prokaryotic Names with Standing in Nomenclature (LPSN; [40]) (Table S3). Then, 7 sequences of the allantoin permease gene were selected for *E. coli* strains (Table S4). 

All sequences were downloaded from the National Center for Biotechnology Information (NCBI) GeneBank database (http://www.ncbi.nlm.nih.gov/, accessed on 21 November 2021). Multiple alignments were performed using MAFFT v7.222 [41] with the default parameters. Conserved regions from aligned sequences for the 16S rRNA gene were used to design the corresponding primers and probes for *Escherichia*/*Shigella* and enterococci. Conversely, the hypervariable zones of 16S rRNA were used to determine *E. faecalis* and *E. faecium*, and the zones of the *ybbW* gene were used for *E. coli*. A total of five pair primers and probes for each group- and species-specific FIBs were obtained through the PrimerQuest tool (Integrated DNA Technologies, Coralville, IA, USA) with default parameters. Secondary structure formation (i.e., primer–primer, primer–probe, or probe–probe interaction) was evaluated using Beacon Designer v7.0 software (Premier Biosoft International, Palo Alto, CA, USA) with the default parameters: GC % ≥ 40–50, Tm = 55 ± 2 °C, and primer length = 18–21 bp. Gibbs free energy changes (ΔG) of <−8 kcal mol^−1^ for hairpin structures and <−9 kcal mol^−1^ for self-dimers and cross-dimers were assumed as exclusion parameters for primer and probe designs, as described in [42,43].

Primers and probes were labelled with fluorophores, as well as internal and external quenchers. Fluorophores labelled at the 5′-end were 6-carboxyfluorescein (6FAM), Hexachloro-Fluorescein-CE Phosphoramidite (HEX), and Cyanine-5 (Cy5). ZEN^TM^ and TAO^TM^ were used as internal quenchers; Iowa Black^®^ F quencher (IABkFQ) and Iowa Black^®^ R quencher Black Hole Quencher^®^ 1 (IAbRQSp) were used as end quenchers at the 3′-end. All primers and probes were validated as described (Table 1) and supplied by Integrated DNA Technologies (IDT, IA, USA).

### 2.2. In Silico Specificity Validation

The SILVA Probe Match and Evaluation Tool (TestProbe 3.0) was used for the validation of 16S rRNA gene primers and probes (Table 1), with 0 mismatches (perfect match). TestProbe calculates the coverages for each taxonomic group in all taxonomies offered in this database [44]. Using the UGENE v33.0 software [45] (Table S5), a second verification was carried out using the primers and probes of enterococci, *E. faecalis*, and *E. faecium* against 68 *Enterococcus* species annotated in the LPSN [40] (Table S5). The primers and probe of the allantoin permease gene (*ybbW*) for *E. coli* were validated through the In Silico PCR Amplification database (http://insilico.ehu.es/PCR/, accessed on 12 December 2021) [46] with UGENE v33.0 software [45].

### 2.3. Study Area and Sample Collection and Preparation

**Figure 1:**
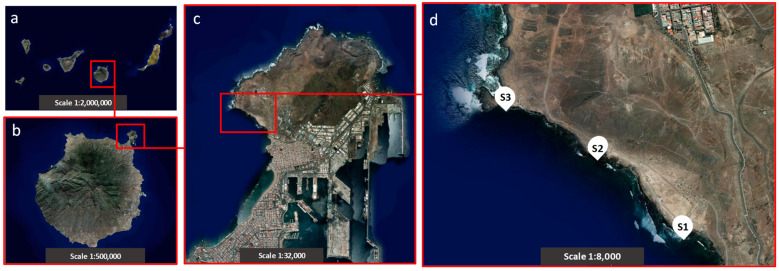
Location of the sampling sites in the bay of *El Confital* (Gran Canaria, Spain): (**a**) the Canary Archipelago, (**b**) the island of Gran Canaria, (**c**) the peninsula of *La Isleta*, and (**d**) the bay of *El Confital*. Sampling site 1 (S1); sampling site 2 (S2); sampling site 3 (S3) (satellite images obtained from Visor IDECanarias, http://visor.grafcan.es/, accessed on 4 February 2024).

The bay of El Confital was selected as the case study. This bay is located in the northeast of the island of Gran Canaria (Spain) (Figure 1). This recreational water zone occupies an area of 634.2 ha [47] and is characterised by a coastal strip, protected from winds, with a maximum depth of approximately 50 m, mostly consisting of sandy bottoms [48]. The coastal area has a volcanic nature and has been affected both by a poor sewerage network discharging into the coastline and the presence of shanties as substandard houses for decades.

Three sampling sites were selected (Figure 1d) according to their exposure to marine currents, namely, the inner site (S1, south), outer site (S3, north), and intermediate site (S2) (Figure 1d). During the two-year sampling period (2022–2023), the bay’s water quality was affected by high concentrations of intestinal enterococci, as reported by Náyade (National Bathing Water Information System, under the jurisdiction of the Ministry of Health, Consumption and Social Welfare of the Government of Spain; http://nayadeciudadano.sanidad.gob.es/, accessed on 10 January 2024) compared to those legally permissible [15]. As a consequence, sampling sites were not influenced by the recent anthropogenic use of the recreational zone.

Seawater samples were collected according to BWD [15]. In short, 2 L of seawater was taken from the water column 30 cm below the surface using sterile polyethylene bottles. The samples were stored in a cool box (4 °C and darkness) and immediately transported to the laboratory for analysis. All were processed within a maximum time interval of 24 h. For analysis, 500 mL of seawater was separately filtered using a 0.45 µm pore size filter (HAWP04700, Millipore, MA, USA). The filters were stored at −20 °C until use.

Marine sediment sampling was carried out by collecting 600 g of the marine substrate (top 4 cm) from the sediment below the water column at each sampling site (Figure 1d) using sterile polyethylene canisters. The canisters were transported in a cool box to the laboratory as described previously. For analysis, 200 g of the substrate was placed in an orbital shaker at 300 rpm for 30 min with sterile artificial seawater (SW, 3.6% (*w*/*v*) Sea Salt mix—Sigma-Aldrich, St. Louis, MO, USA—in distilled water) at a ratio of 1:1. Supernatants were filtered individually, as described above.

Sampling was performed in triplicate for each seawater and sediment sample, sampling site, and time period.

### 2.4. DNA Extraction

Filters were individually homogenised in liquid nitrogen, and each one was individually placed in microtubes for the extraction of environmental DNA (eDNA). eDNA extraction was performed according to the CTAB method described in [49] and modified in [50].

In detail, the extraction buffer was prepared using CTAB 2% (*w*/*v*), PVPP 0.1% (*w*/*v*), TRIS-HCl 100 mM pH 8.6, SDS 10%, EDTA 0.5 M pH 8, NaCl 4 M, and β-Mercaptoethanol 2% (*v*/*v*). Next, 800 µL of extraction buffer was added to each sample. The samples were then kept in a bath at 65 °C for 1 h and gently mixed via inversion three times, in addition to the incubation step. Later, 800 µL of CIA (chloroform–isoamyl alcohol, 24:1 (*v*/*v*)) was added, and the samples were centrifuged for 20 min at 3000 rpm in a VWR Micro Star 17R centrifuge (VWR International Eurolab, BCN, Barcelona, Spain). Successive washes in CIA and centrifugations were carried out until the supernatant became whitish. To continue, 2/3 of isopropyl alcohol at −20 °C was added, and centrifugation at 15,000× *g* for 30 min was carried out. Afterwards, the isopropyl alcohol was removed, and 20 µL of ethanol (80%) was added. Finally, the samples were centrifuged at 15,000× *g* for 5 min. The supernatant was discarded, and the pellets were resuspended in 15 µL of DNase-Free ddH_2_O and stored at 4 °C until used. The yield and purity of genomic DNA were calculated from the A260/A280 ratio measured using a NanoDrop spectrophotometer (ThemoFisher Scientific, Waltham, MA, USA). DNA extraction was performed in triplicate for each sample type (seawater and marine sediment), sampling site, and period.

### 2.5. Multiplex RT qPCR Amplification

#### 2.5.1. Construction of Standard Curves for Gene Copy Number Determination

Standard curves were obtained with respect to the pure strains of *Enterococcus faecium* (WDCM 00010) and *Enterococcus faecalis* (WDCM 00009), with the latter being representative of group-specific enterococci [51]. Moreover, curves were designed using *Escherichia coli* (WDCM 00012) for group-specific *Escherichia*/*Shigella* and species-specific *E. coli*. Bacterium strains were supplied by the Spanish Type Culture Collection of the University of Valencia (CECT-UV), with a concentration of 10^10^ CFU mL^−1^. All are catalogued as reference strains to be used in water quality controls regulated by ISO standards (*E. faecalis* WDCM 00009 used in Spanish Norm (UNE)-European Standard (EN) International Organization for Standardization (ISO) such as UNE-EN ISO7899-2:2001; *E. faecium* WDCM 00010 used in UNE-EN ISO/TS 11133-1:2009; *E. coli* WDCM 00012 used in UNE-EN ISO 9308-1:2014).

Bacterium strain DNA was individually extracted and quantified, as previously described for eDNA, using 200 µL of each strain type. The strain’s DNA was then diluted via a 10-fold serial dilution. In total, 2 μL of each dilution was used for each PCR reaction mixture containing 5 μL of 2×PrimeTime Gene Expression Master Mix (IDT), 0.4 μL of forward and reverse primers (400 nM) (Table 1), and 0.2 µL of TaqMan probe (200 nM) up to a final 10 μL volume. Amplification was performed at 95 °C for 3 min, followed by 45 cycles of a two-step PCR programme consisting of 95 °C for 15 s and 55 °C for 60 s, using a Bio-Rad CFX96 instrument with a CFX96™ Real-Time PCR Detection System (Bio-Rad, Hercules, CA, USA). The amplification value was recorded as the quantification cycle (C_q_). This means that the cycle in which the fluorescence of the PCR product was detected is above the quantification cycle (C_q_) according to MIQE guidelines [52]. Data acquisition and analyses were performed using Bio-Rad CFX Maestro v1.0 software (Bio-Rad).

Quantification cycle (C_q_) values were plotted against the logarithm of the corresponding gene copy number of each 10-fold serial dilution using the IBM SPSS Statistics 27 statistical programme (IBM SPSS Inc., Armonk, NY, USA). A standard curve for each pure strain was generated. The amplification efficiency (E) of each standard curve was calculated using the equation described as follows [53]: E = 10(−1/slope)^−1^. The limit of detection (LOD) was also determined for each standard curve. The corresponding gene copy number was calculated using Equation (1) described as follows [54]:

(1) $$gene\;copy\;number=\frac{6.02\times 10^{23}\;\left(copy\;{mol}^{-1}\right)\times DNA\;amount\;(g)}{DNA\;length\;\left(bp\right)\times 660\;(g\;{mol}^{-1}{bp}^{-1})}$$

where 6022 × 10^23^ copy mol^−1^ = Avogadro’s number, the DNA amount of the template, and DNA length of the amplicon in base pairs; and 660 g mol^−1^ bp^−1^ = the average mass of one base pair.

Gene copy numbers were recalculated according to the operon number of each pure strain, namely, 4 copies of *rrn* operons correspond to *E. faecalis*; 6 copies of *rrn* operons correspond to *E. faecium*; and 7 copies of *rrn* operons correspond to *E. coli*. The number of rrn operons of each strain type was obtained from the Ribosomal RNA Operon Copy Number Database (*rrn*DB; https://rrndb.umms.med.umich.edu/, accessed on 25 January 2022). The final gene copy number was obtained by dividing the resulting number of 16S rRNA gene copies by the average number of *rrn* operons for each bacterial species. Pure DNA strains were isolated and amplified using three samples each.

#### 2.5.2. Quantification of Faecal Indicator Bacteria

Two instances of multiplex RT qPCR were set for amplification according to the group-specific enterococci (16S rRNA) and *Escherichia*/*Shigella* (16S rRNA) targets and species-specific *E. faecalis* (16S rRNA), *E. faecium* (16S rRNA), and *E. coli* (*ybbW*) targets. All multiplex RT qPCR assays were run with appropriate controls, such as non-template controls (NTCs) and positive controls performed with DNA from the bacterial strain type corresponding to each assay. All PCR reactions were performed as described for bacterial strain DNA using three pooled samples each. Absolute quantifications were expressed as the target gene copy numbers of group- or species-specific FIBs for water (gene copy number per 100 mL^−1^ of seawater) and sediment (gene copy number per 100 g^−1^ of marine sediment) samples by relating their C_q_ value to the standard curves of each one pure strain.

### 2.6. Data Analysis

Data analysis was carried out in two seasons (i.e., winter and spring) as non-differences between the two years were detected. One-way ANOVA followed by Tukey’s post hoc test (HSD) was carried out through IBM^®^ SPSS^®^ Statistics v27.0 software (IBM, NY, USA) for comparing significant differences (*p* ≤ 0.05; 95% confidence interval) between the average number of gene copies reported in seawater and those of marine sediment; between the seasons for 2 years; and between FIB types. A Venn diagram was created to visualise the results of the in silico validation of enterococci assays using Venny 2.1.0 software (BioinfoGP Service, CNB-CSIC, Spain) [55].

## 3. Results

### 3.1. In Silico Validation for Group- and Species-Specific

The highest coverage out of the five pairs of primers and probes designed for the enterococci group was achieved by primer pair EF399F and EF484R and its ProbeEN. The primer pair and probe (henceforth RT qPCR assay) were amplified for 1582 species from the Bacteria domain and 947 species from *Enterococcus* (Table 2).

The primers and probes for *E. faecalis* and *E. faecium* with the highest coverage are EF1203F, EF1305R, ProbeEf and EFium96F, EFium225R, ProbeEFium, respectively. The *E. faecalis* RT qPCR assay shows that out of 302 sequences of the Bacteria domain from the SILVA database, 292 belong to the genus *Enterococcus*. Furthermore, of these 292 sequences, 203 were specifically identified as *E. faecalis*, and the remaining were identified as *Enterococcus* sp. In contrast, the *E. faecium* RT qPCR assay reveals that 179 sequences are *Enterococcus*, 129 are *E. faecium*, and the remaining are *Enterococcus* sp. (Table 2). The second verification was carried out for enterococci, *E. faecalis*, and *E. faecium*, which revealed that out of 68 *Enterococcus* species annotated in the LPSN [40] (Table S5), 16 were confirmed for primers and probes through the UGENE software [45].

**Figure 2:**
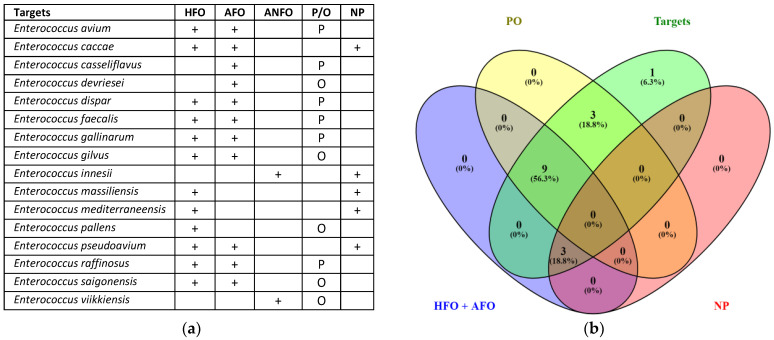
*Enterococcus* species annotated in the List of Prokaryotic Names with Standing in Nomenclature (LPSN) [40] and in silico amplified with the group-specific primers and probes designed in this work using UGENE v33.0 software [45]. (**a**) Species assignation according to the origin and clinical interest of *Enterococcus* species; (**b**) Venn diagram of *Enterococcus* species retrieved from LPSN. HFO: human faecal origin; AFO: animal faecal origin; ANFO: animal non-faecal origin; P/O: pathogenic or opportunistic species; NP: non-pathogenic species; +: shows the positive assignment of the target.

Furthermore, to assign the origin and examine the clinical interest for these species—namely, human or animal faecal origin (HFO; AFO, respectively); animal non-faecal origin (ANFO); pathogenic or opportunistic species (P/O); non-pathogenic species (NP)—a classification was made based on information retrieved from NCBI and LPSN (Figure 2).

The results show that 15 *Enterococcus* species (i.e., 93.9%; Figure 2b) from UGENE were annotated as faecal and with clinical interest. In contrast, *Enterococcus innesii* (Figure 2a) was classified as an animal of non-faecal origin and a non-pathogenic species. In addition, RT qPCR assays for *E. faecalis* and *E. faecium*, as representative intestinal enterococci, confirmed that each species was differentially amplified from other deposited species (Table S5).

The highest coverage for assigning the taxon to the group-specific *Escherichia*/*Shigella* was obtained with EC501F, EC604R, and ProbeES. The results confirmed that 76% of amplified sequences covered the *Escherichia*/*Shigella* group, namely, 2124 belonged to the *Escherichia*/*Shigella* sequences against 2153 of the Bacteria domain sequences (Table 3).

Regarding the species-specific group, an in silico amplification using the single-copy gene *ybbW* revealed *E. coli* strains. Non-amplification was realised for other species of *Escherichia* and *Shigella* strains (Table S6).

### 3.2. Multiplex RT qPCR Amplification

#### 3.2.1. Linearity and Analytical Sensitivity of the Multiplex RT qPCR Assays

**Figure 3:**
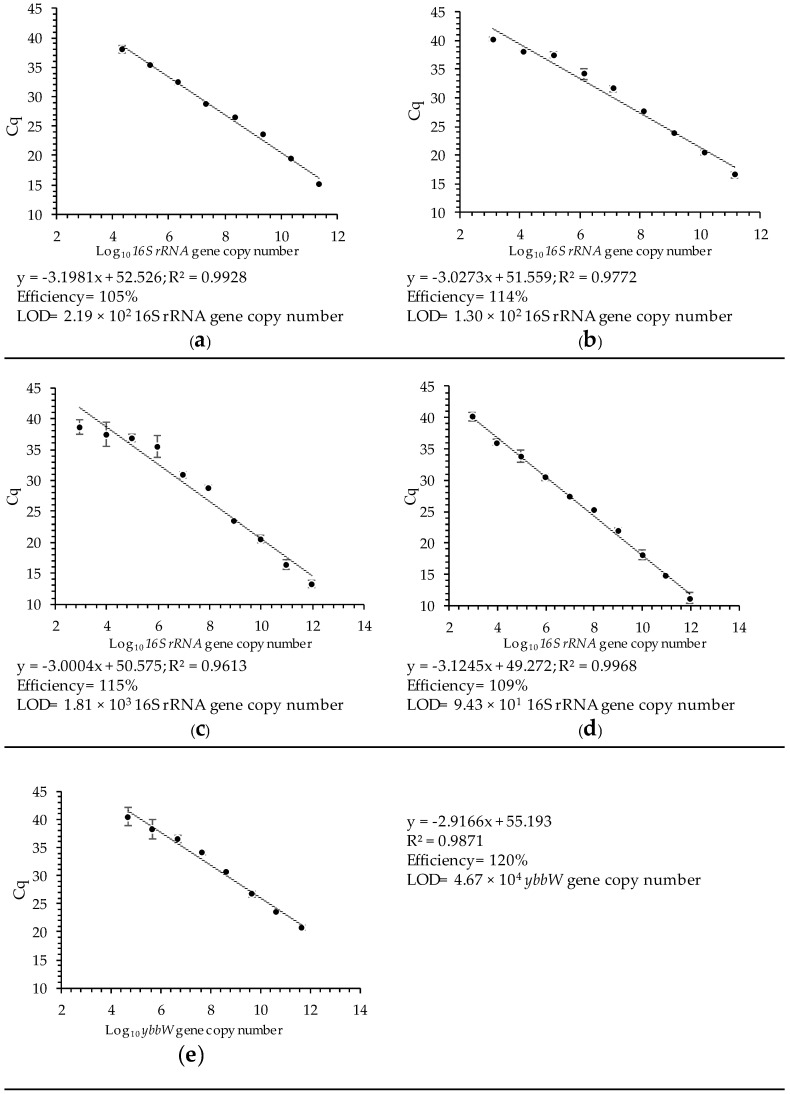
Correlation between the log_10_ gene copy number and quantification cycle (C_q_) values with indications of efficiency and the limit of detection of (**a**) enterococci, (**b**) *Escherichia*/*Shigella*, (**c**) *E. faecalis*, (**d**) *E. faecium*, and (**e**) *E. coli*. RT qPCR efficiency was calculated according to [53] (*n* = 3).

The RT qPCR assays for group-specific enterococci and *Escherichia*/*Shigella* show amplification efficiencies of 105 and 114% (R^2^ ≥ 0.970 in both cases) and linearity for the copy number of the 16S rRNA gene against C_q_, respectively (Figure 3 and Figure S1a,b). The limit of detection (LOD), defined as the gene copy number (or lowest dilution) at which a C_q_ value was obtained, is 2.19 × 10^2^ and 1.30 × 10^2^ for enterococci and *Escherichia*/*Shigella*, respectively (Figure 3a,b).

The species-specific RT qPCR assays show efficiencies of 115, 109, and 120% for *E. faecalis*, *E. faecium*, and *E. coli*, respectively (R^2^ ≥ 0.960; Figure 3 and Figure S1c–e). The limits of detection are 1.81 × 10^3^ gene copy numbers for *E. faecalis*, 9.43 × 10^1^ gene copy numbers for *E. faecium*, and 4.67 × 10^4^ gene copy numbers for *E. coli*. (Figure 3c–e).

#### 3.2.2. Quantification of Faecal Indicator Bacteria

The 16S rRNA copy number of group-specific enterococci is 1 to 2 times higher than that for *Eschechirichia*/*Shigella* both in seawater and marine sediments (Table 4). Moreover, a higher copy number for group-specific enterococci and *Escherichia*/*Shigella* is obtained in sediments compared to seawater (Table 4). Significant differences (*p* < 0.05; Table 4) are reported in sediments during winter compared to spring.

Non-copies for the allantoin transporter-encoding gene (*ybbW*) of *E. coli* are detected via multiplex RT qPCR. Conversely, the expression of the *ybbW* gene, through a singleplex, was detected in seawater during two seasons (winter: 4.9 ± 1.5 × 10^5^ gene copies at 100 mL^−1^; spring: 2.1 ± 0.2 × 10^6^ gene copies at 100 mL^−1^) at the northern station (S3) in 2023.

The *Enterococcus faecalis* gene copy number is significantly higher (*p* < 0.05) in sampling site S2 during spring (1.5 × 10^6^ ± 0.1 16S rRNA copies 100 mL^−1^) than in the other sampling sites (S1 and S3) (Table 5).

Regarding marine sediment, *E. faecalis* is reported to be 1 order of magnitude higher in S3 than in the inner sampling sites (S2) during the winter (Table 5). Moreover, the contribution of *E. faecalis* to the enterococci group is non-relevant in marine sediment as it ranges from 2.0 to 4.2% of the total gene copy number for the S1 and S2 sampling sites during spring. In contrast, the contribution reaches 79.80% and 65.38% at S3 during winter and spring, respectively.

The detection of *E. faecium*, through gene copy numbers, ranges from 0 copies to 1 or 3 orders of magnitude lower than *E. faecalis* depending on the season and whether seawater and marine sediment were analysed (Table 5).

## 4. Discussion

### 4.1. In Silico Validation for Group- and Species-Specific Data

This study provides insights into the molecular characterisation of the faecal indicator bacteria *Escherichia coli* and intestinal enterococci, which are microbiological reference parameters in the European Bathing Water Quality Assessment Regulation [15]. A molecular approach allows for the specific quantification of strains and a determination at low concentrations as it is highly sensitive and produces rapid diagnoses as processing times are reduced [16,17,18].

The first benchmark was to molecularly identify group- and species-specific *Escherichia coli* via the 16S rRNA gene, which represents the standard molecular marker of prokaryotes [23,26]. While *E. coli* has been determined through culture-dependent procedures, the identification via 16S rRNA reveals that *E. coli* encompassed the identification of the *Escherichia* and *Shigella* genera. A high specificity of the primers and probe (Table 1) was obtained for *Escherichia* and *Shigella* sequences through in silico amplification as 76% of the sequences matched with the taxon sequences deposited in the SILVA database [44] with respect to *Escherichia*/*Shigella* (Table 3). It is known that *Escherichia* and *Shigella* share a high number of genome sequences: i.e., 98.4% sequences of both bacteria have a close similarity [56,57,58]. Moreover, evidence shows that the taxonomic boundaries between genera *Escherichia* and *Shigella* are scattered [25,57,58,59]. It is worth mentioning that the 16S rRNA gene sequence has a similarity of > 99% when *E. coli* and *Shigella* spp. are compared [60].

Different complete and partial gene sequences have been proposed for the molecular identification of this species [20,61]. Among them, the *ybbW* gene encoding a putative allantoin permease revealed high specificity with respect to *E. coli* (100%) and distinguished the other species of the genus *Escherichia* from *Shigella* spp. [28,29,30,31,62]. Additionally, the in silico PCR Amplification database and UGENE software showed that the *ybbW* gene amplified 95% of *E. coli* sequences deposited in databases, but no amplification was reported for other species of the *Escherichia* or *Shigella* genera (Table S6).

Concerning *Enterococcus*, the 16S rRNA results showed reliable information at the group level. In total, 61.1% of the sequences matched with the target taxon sequences (*Enterococcus*) were deposited in the SILVA database (Table 2). Meanwhile, 93.9% of the species of *Enterococcus* were annotated and recognised to be of faecal origin and had a clinical interest (Figure 2). The difference between coverage percentages was interpreted as some *Enterococcus* spp. having an undetermined faecal origin and clinical interest (Figure 2; Table S5). Moreover, several issues are reported for the molecular identification of intestinal enterococci due to the FIB reference in the BWD [15] through the *Enterococcus* genus [63,64,65,66]. The term intestinal enterococci has largely been interchangeable with enterococci, thus addressing the non-accurate identification of FIB references [67]. Furthermore, intestinal enterococci species such as *Enterococcus faecium* and *Enterococcus faecalis* have been reported as predominant in the human gastrointestinal tract [32,33,35], and they are responsible for most enterococcal infections in humans [36,37]. In this sense, this work reports species-specific assays for *E. faecium* and *E. faecalis* with 100% specificity (Table 2 and Table S5).

### 4.2. Quantification of Faecal Indicator Bacteria

The quantification of FIBs, through multiplex RT qPCR, has been validated as high values of linear correlation (R^2^ ≥ 0.960) are obtained. Moreover, amplification efficiency values ranging from 105 to 120% are appropriate [20,68].

A higher 16S rRNA gene copy number is reported for the enterococci group compared to the *Escherichia/Shigella* group (Table 4). In addition, the quantification of FIBs was higher in marine sediments compared to seawater (Table 4 and Table 5). These results point to the outstanding contribution of sediment to FIB determination, as explained below. Moreover, non-copies for the allantoin transporter-encoding gene *(ybbW*) of *E. coli* were detected via multiplex RT qPCR. Multiplex non-amplifications for the allantoin gene still have to be solved as masked signals can continue to occur [24,69] compared to the positive amplifications obtained through singleplex assays.

Significant differences with respect to the 16S rRNA gene copies of enterococci were observed in sampling sites S1 and S2 for seawater during both seasons (Table 4). Conversely, sampling site S3 reports significant concentrations of the enterococci group in marine sediments during spring. Moreover, it was significant that S1 and S2 reached higher concentrations of 3 to 4 orders of magnitude during winter compared to those in spring with respect to sediments (Table 4).

While *E. faecalis* exhibited coincident spatial and temporal trends with respect to the enterococci group in seawater, the contribution of *E. faecalis* to the enterococci group was not relevant in seawater (<2.38% total gene copy number; Table 5). Likewise, this contribution was also low in marine sediments with a range between 2.0% and 4.2% of the total gene copy number for the S1 and S2 sampling sites during spring (Table 5). Given that *E. faecium* was always reported to be 1–3 orders of magnitude below *E. faecalis*, other enterococci of faecal origin are contributing to this group-specific FIB.

Notably, the enterococci group is supported by *E. faecalis* in a range from 65.38 to 79.80% in marine sediments at S3 (Table 5). The fact that S3 corresponded to an exposed zone with marine dynamics allows us to infer that the processes of bacterial naturalisation could be occurring in this area. This could be supported if the geological (volcanic) features of the studied area were considered. This means that the porous substrates with submerged volcanic tubes could be the pioneers of microbial colonisation, explaining biofilm formation and bacteria anchoring in sediments [70,71]. Naturalised enterococci could create biofilms and thus proliferate under a variety of conditions [72]. In this regard, the contribution of *E. faecalis* could be overestimated as *E. faecalis* from both naturalised bacterium pools and recent bacterium pollution are being considered. Nonetheless, it is worth mentioning that naturalised bacterium pools are a relevant source of environmentally persistent pathogens, with a potential impact on human health [73,74,75]. In this scenery disparities in FIB concentration between sampling sites, seasons and matrices allow us to infer that increment in enterococci is due to the removal of sediments by coastal wave dynamic in winter while an input of non-human faecal material due to the settlement of birds in the area in spring could contribute to this FIB budget (Table 4). The presence of *E. coli* and *Enterococcus* spp. in seagull guano deposits has been reported in higher concentrations [76] and, in turn, associated with beach sediment. Inoculation waters were also reported when the sediments were disturbed by waves or swimmers [77]. Hence, sediment monitoring is recommended for inclusion in the valuation of marine coastal system quality. Results point out that approaches for the identification of sources and how some strains appear to be associated with certain host sources have to be also monitored.

In summary, the molecular approach allows the identification of the *Escherichia/Shigella* group and enterococci of faecal origin through the 16S rRNA gene. Species-specific assays also reveal high specificity for *E. faecium* and *E. faecalis*. Considering the sanitary aspects, the authors propose sediment analysis for quality assessment of marine coastal systems.

## Figures and Tables

**Table 1:** Sequences of primers and TaqMan probes designed, validated, and used in the multiplex RT qPCR assays carried out in this study.

Target	FIB	Gene	Primers and Probes	Sequence (5′–3′) ^a^	Product Size (bp)
Group-specific	Enterococci	16S rRNA	EF399F EF484R ProbeEN	F: GCTCGTGTCGTGAGATGTT R: CGCTAGAGTGCCCAACTAAA P: [HEX]-AACAATAAG-[ZEN]-GGTTGCGCTCGTTGC-[IABkFQ]	86
	*Escherichia/Shigella*	16S rRNA	EC501F EC604R ProbeES	F: TCGGAATTACTGGGCGTAAAG R: GACTCAAGCTTGCCAGTATCA P: [6FAM]ACGCAGGCG-[ZEN]-GTTTGTTAAGTCAGA-[IABkFQ]	104
Specie-specific	*Enterococcus faecalis*	16S rRNA	EF1203F EF1305R ProbeEf	F: CTTATGACCTGGGCTACACAC R: CTGCAATCCGAACTGAGAGAA P: [Cy5]-ACCGCGAGG-[TAO]-TCATGCAAATCTCTT-[3IAbRQSp]	103
	*Enterococcus faecium*	16S rRNA	EFium96F EFium225R ProbeEFium	F: TGGCGAACGGGTGAGTAA R: TCCATCCATCAGCGACAC P: [6FAM]-CAAAACCGC-[ZEN]-ATGGTTTTGATTTGAAAGGCG-[3IABkFQ]	140
	*E. coli*	*ybbW*	ybbWF ybbWR ProbeybbW	F: GCAAAATCTGGCCGGGAT R: AATCGCCCAAATCGCCA P: [HEX]-CACTGCCAT-[ZEN]-TCTTAACCCGTGCATC-[3IABkFQ]	203

^a^ F, R, and P indicate forward primers, reverse primers, and probes, respectively.

**Table 2:** In silico taxon-specific results of group- and species-specific primers and TaqMan probes designed for enterococci and *E. faecalis* and *E. faecium*, respectively.

		Percentage of Coverage in the Target Taxon (%) ^a^ % (Number of Sequences Matched/Total Number of Taxon Sequences Deposited)		
	Target Assay Primers and Probes	Enterococci (Group-Specific) EF399F, EF484R, ProbeEN	*Enterococcus faecalis* (Species-Specific) EF1203F, EF1305R, ProbeEf	*Enterococcus faecium* (Species-Specific) EFium96F, Fium225R, ProbeEFium
Target Taxon				
Bacteria Domain		0.4 (1582/381,535)	0.1 (302/381,535)	0.0 (180/381,535)
Desulfobacterota		0.4 (1/7818)	0.0 (0/7818)	0.0 (0/7818)
Proteobacteria		0.0 (22/109,146)	0.0 (1/109,146)	0.0 (0/109,146)
**Firmicutes**		**1.6 (1559/100,538)**	**0.3 (301/100,538)**	**0.2 (180/100,538)**
Dethiobacteria		1.5 (2/137)	0.0 (0/137)	0.0 (0/137)
Clostridia		0.1 (32/50,639)	0.0 (2/50,639)	0.0 (0/50,639)
**Bacilli**		**3.6 (1525/42,707)**	**0.7 (299/42,707)**	**0.7 (180/42,707)**
**Lactobacillales**		**12.0 (1525/13,176)**	**19.0 (295/13,176)**	**1.4 (180/13,176)**
Aerococcaceae		0.8 (2/264)	0.0 (0/264)	0.0 (0/264)
Streptococcaceae		9.7 (520/5371)	0.0 (1/5371)	0.0 (1/5371)
Lactobacillaceae		0.2 (11/4739)	0.0 (2/4739)	0.0 (0/4739)
Vagococcaceae		78.3 (36/46)	0.0 (0/46)	0.0 (0/46)
**Enterococcaceae**		**59.8 (956/1600)**	**18.4 (292/1600)**	**11.3 (179/1600)**
*Melissococcus*		100.0 (9/9)	0.0 (0/9)	0.0 (0/9)
*Enterococcus*		**61.1 (947/1551)**	**19.0 (292/1551)**	**11.7 (179/1551)**
*Enterococcus faecalis*		-	**80.2 (203/253)**	0.8 (3/253)
*Enterococcus faecium*		-	0.4 (1/261)	**49.4 (129/261)**
*Enterococcus* sp.		-	16.0 (28/175)	13.1 (23/175)
Uncultured *Enterococcus* sp.		-	23.1 (52/225)	7.6 (17/225)

Taxonomic levels corresponding to the target FIBs (enterococci, *E. faecalis*, and *E. faecium*) are highlighted in bold; ^a^ percentage of sequence coverage in the target group based on the SILVA 138 SSU Ref NR database (https://www.arb-silva.de/search/testprobe/, accessed on 15 May 2023), released in May 2023.

**Table 3:** In silico taxon-specific results of group-specific primers and probes designed for *Escherichia/Shigella*.

Target Taxon	Percentage of Coverage in the Target Taxon (%) ^a^ % (Number of Sequences Matched/Total Number of Taxon Sequences Deposited)
Bacteria Domain	0.6 (2153/381,535)
Firmicutes	0.0 (5/100,538)
**Proteobacteria**	**2.0 (2147/109,146)**
Burkholderiales	0.0 (2/20,421)
Pseudomonadales	0.0 (2/17,709)
**Gammaproteobacteria**	**2.9 (2147/75,026)**
**Enterobacterales**	**9.3 (2143/23,028)**
Erwiniaceae	0.1 (1/1233)
Pectobacteriaceae	0.2 (1/433)
**Enterobacteriaceae**	**27.0 (2141/7969)**
*Citrobacter*	0.2 (1/516)
*Enterobacter*	0.2 (3/1670)
*Klebsiella*	0.4 (5/1177)
*Salmonella*	0.8 (8/952)
***Escherichia/Shigella***	**76.0 (2124/2807)**
***Escherichia coli***	**98.2 (1019/1038)**
***Escherichia fergusonii***	**95.7 (22/23)**
***Escherichia albertii***	**27.3 (6/22)**
***Shigella flexneri***	**100.0 (53/53)**
***Shigella boydii***	**100.0 (24/24)**
***Shigella dysenteriae***	**87.2 (34/39)**
***Shigella sonnei***	**91.4 (32/35)**
*Escherichia* sp.	94.4 (34/36)
*Shigella* sp.	90.9 (10/11)
*Escherichia/Shigella* ^b^	68.8 (242/352)
Uncultured *Escherichia* sp.	28.6 (6/21)
Uncultured *Shigella* sp.	70.0 (14/20)
Uncultured *Escherichia/Shigella* ^c^	59.6 (628/1054)

Taxonomic levels corresponding to the target FIB *Escherichia*/*Shigella* are highlighted in bold. ^a^ Percentage of sequence coverage in the target group based on the SILVA 138 SSU Ref NR database (https://www.arb-silva.de/search/testprobe/, accessed on 27 May 2023). ^b^ Unidentified species belonging to the taxon. ^c^ Unidentified and uncultured species belonging to the taxon.

**Table 4:** Absolute quantification of the 16S rRNA gene for group-specific enterococci and *Escherichia/Shigella* in seawater and marine sediments through multiplex RT qPCR assays. Data are expressed as the mean of gene copies (in 100 mL^−1^ of seawater or 100 g^−1^ of marine sediments) ± SD (in brackets). **a**: Indicates significant differences between sampling sites (*p* < 0.05). **b**: Indicates significant differences between seasons (*p* < 0.05). **c**: Indicates significant differences between the FIB groups (enterococci and *Escherichia/Shigella*) (*p* < 0.05) (*n* = 6).

Sampling		Seawater (Gene Copy Number in 100 mL^−1^)		Marine Sediments (Gene Copy Number in 100 g^−1^)	
Period	Site	Enterococci	*Escherichia/Shigella*	Enterococci	*Escherichia/Shigella*
Winter	S1	2.4 (1.7) × 10^7^ **c**	7.7 (3.4) × 10^5^ **c**	8.2 (0.2) × 10^8^	2.4 (1.8) × 10^5^ **a;c**
	S2	1.2 (0.6) × 10^7^	2.7 (0.9) × 10^6^ **a**	7.5 (0.3) × 10^9^	2.1 (0.9) × 10^7^ **c**
	S3	1.5 (0.1) × 10^7^ **c**	3.8 (3.1) × 10^4^ **a**	9.9 (0.7) × 10^5^ **a;c**	3.0 (2.2) × 10^7^ **c**
Spring	S1	1.8 (0.2) × 10^7^	1.8 (0.6) × 10^6^	4.5 (1.7) × 10^5^ **b**	1.2 (1.0) × 10^5^ **a**
	S2	6.3 (1.0) × 10^7^	2.4 (0.8) × 10^6^	1.8 (1.3) × 10^5^ **b**	9.2 (2.6) × 10^4^ **a;b**
	S3	2.1 (0.5) × 10^7^	2.0 (2.7) × 10^4^ **a;c**	1.3 (1.0) × 10^7^ **a;b**	2.4 (0.4) × 10^6^ **b**

**Table 5:** Absolute quantification of the 16S rRNA gene for species-specific *Enterococcus faecalis* and *Enterococcus faecium* in seawater and marine sediments through multiplex RT qPCR assays. Data are expressed as the mean of gene copies (in 100 mL^−1^ of seawater or 100 g^−1^ of marine sediments) ± SD (in brackets). The percentage in brackets corresponds to the contribution of the *Enterococcus faecalis* species to the total enterococci group. No data means data are below the limit of quantification. **a**: Indicates significant differences between sampling sites (*p* < 0.05). **b**: Indicates significant differences between seasons (*p* < 0.05). **c**: Indicates significant differences between the FIB groups (enterococci and *Escherichia*/*Shigella*) (*p* < 0.05) (*n* = 6).

Sampling	Seawater (Gene Copy Number in 100 mL^−1^)			Marine Sediments (Gene Copy Number in 100 g^−1^)	
Period	Station	*Enterococcus faecalis*	*Enterococcus faecium*	*Enterococcus faecalis*	*Enterococcus faecium*
Winter	S1	4.7 (6.2) × 10^4^ (0.20%)	No data	5.4 (3.0) × 10^5^ (0.07%)	No data
	S2	4.3 (2.7) × 10^4^ (0.36%)	1.0 (1.7) × 10^3^	3.6 (0.2) × 10^4^ (0.001%)	No data
	S3	3.0 (3.2) × 10^4^ (0.20%)	2.3 (0.7) × 10^3^	7.9 (2.6) × 10^5^ (79.80%) **c**	3.6 (0.8) × 10^2^ **b**
Spring	S1	1.5 (0.3) × 10^4^ (0.08%)	No data	1.9 (1.9) × 10^4^ (4.20%)	4.0 (1.6) × 10^3^ **b**
	S2	1.5 (0.1) × 10^6^ (2.38%) **a;b**	No data	4.0 (0.6) × 10^3^ (2.20%)	1.4 (0.1) × 10^2^ **b**
	S3	1.1 (0.4) × 10^4^ (0.05%)	1.1 (1.8) × 10^3^	8.5 (1.2) × 10^6^ (65.39%) **a**	No data

## Data Availability

The original contributions presented in the study are included in the article/Supplementary Materials, further inquiries can be directed to the corresponding authors.

## Supplementary Materials

The following supporting information can be downloaded at https://www.mdpi.com/article/10.3390/microorganisms12061223/s1. Table S1. Sequences of the 16S rRNA gene of *Escherichia* and *Shigella* species used to design group-specific primers and the *Escherichia*/*Shigella* TaqMan probe. Table S2. Sequences of the *16S rRNA gene* of *Enterococcus* species used to design group-specific primers and TaqMan probes for enterococci. Table S3. Sequences of the *16S rRNA* gene of *Enterococcus* species used for the design of species-specific primers and TaqMan probes of *E. faecalis* and *E. faecium*. Table S4. Sequences of the allantoin transporter-encoding gene (*ybbW*) of *Escherichia coli* strains used for the design of species-specific primers and *E. coli* TaqMan probes. Table S5. Results of the in silico amplifications of the 16S rRNA gene performed with the primers and TaqMan probes of group- and species-specific enterococci, *E. faecalis*, and *E. faecium* using a dataset built with the sequences of 68 *Enterococcus* species and accepted by the LPSN (List of Prokaryotic Names with Standing in Nomenclature [39]) as a template, and the results were obtained in the UGENE v33.0 software [44]. Table S6. Results of the in silico amplification of the allantoin transporter-encoding gene (*ybbW*) for the species-specific *E. coli* assay performed in the UGENE v33.0 software [44] and the In Silico PCR Amplification tool [45]. Figure S1. C_q_ determination for the 16S rRNA gene of group-specific (a) enterococci and (b) *Escherichia/Shigella* FIB and species-specific (c) *Enterococcus faecalis* and (d) *Enterococcus faecium*; and for (e) the allantoin transporter-encoding gene (*ybbW*) of the species-specific determination of *Escherichia coli*.
