# Supplementary material for: Development of Multiplex RT qPCR Assays for Simultaneous Detection and Quantification of Faecal Indicator Bacteria in Bathing Recreational Waters
Source: Microorganisms. 2024 Jun 18;12(6):1223. doi: 10.3390/microorganisms12061223 (PMC11205496; doi:10.3390/microorganisms12061223)
Supplement: Supplementary file 1 [file microorganisms-12-01223-s001.zip › Table S1.pdf]

Table S1. Sequences of the *16S rRNA* gene of *Escherichia* and *Shigella* species used to design group-specific primers and the *Escherichia*/*Shigella* TaqMan probe.

**Species and strains**	**NCBI accession number**	**Host[*] / Source**
Escherichia coli AE1-2	AB269763	H / Faeces
Escherichia coli O157:H7	AY513502	H / CS
Escherichia coli XJ133-127-1NF1	JX975415	H / CS
Escherichia coli K-12 / MG1655	NC_000913[a]	-
Escherichia coli K-12 / W3110	NC_007779[a]	-
Escherichia fergusonii 190311L245	MT225668	A
Escherichia marmotae HT073016	MH972185	A / Sewage
Shigella dysenteriae FBD015	EU009186	H / CS
Shigella flexneri GH24	KC887964	H / Faeces
Shigella sonnei CECT 4887	NR_104826	-
Shigella boydii P288	NR_104901	-

[*]H: Human; E: Environment; An: Animal; CS: Clinical Sample.

[a] Whole genome sequence was used (*16S rRNA* gene sequences not available).
