# Supplementary material for: Development of Multiplex RT qPCR Assays for Simultaneous Detection and Quantification of Faecal Indicator Bacteria in Bathing Recreational Waters
Source: Microorganisms. 2024 Jun 18;12(6):1223. doi: 10.3390/microorganisms12061223 (PMC11205496; doi:10.3390/microorganisms12061223)
Supplement: Supplementary file 1 [file microorganisms-12-01223-s001.zip › Table S2.pdf]

Table S2. Sequences of the *16S rRNA gene* of *Enterococcus* species used to design group-specific primers and TaqMan probes for enterococci.

**Species and strains**	**NCBI accession number**	**Host[*] / Source**
Enterococcus avium E16	MK322649	H / CS
Enterococcus caccae 2215-02	NR_043285	H / Faeces
Enterococcus casseliflavus N1-M-4	OK271983	H / Faeces
Enterococcus devriesei ESW6	OQ692578	A / Faeces
Enterococcus dispar MG4619	ON631303	H / Faeces
Enterococcus faecalis HASOB12b	MH291406	H / Faeces
Enterococcus gallinarum CKY	AB269767	H / Faeces
Enterococcus gilvus PQ1	NR_115121	H / Faeces
Enterococcus massiliensis AM1	NR_144723	H / Faeces
Enterococcus mediterraneensis Marseille-P4358	NR_179501	H / Faeces
Enterococcus pallens PQ2	NR_115122	H / Faeces
Enterococcus pseudoavium Hana51	MN399952	An / Faeces
Enterococcus raffinosus P6	DQ223885	An / Faeces
Enterococcus saigonensis HM14	MN401131	An / Faeces

[*]H: Human; An: Animal; CS: Clinical Sample
