# Supplementary material for: Development of Multiplex RT qPCR Assays for Simultaneous Detection and Quantification of Faecal Indicator Bacteria in Bathing Recreational Waters
Source: Microorganisms. 2024 Jun 18;12(6):1223. doi: 10.3390/microorganisms12061223 (PMC11205496; doi:10.3390/microorganisms12061223)
Supplement: Supplementary file 1 [file microorganisms-12-01223-s001.zip › Table S3.pdf]

Table S3. Sequences of the *16S rRNA* gene of *Enterococcus* species used for the design of species-specific primers and TaqMan probes of *E. faecalis* and *E. faecium*.

**Species and strains**	**NCBI accession number**
Enterococcus alcedinis L34	NR_109727
Enterococcus alishanensis ALS3	NR_178249
Enterococcus aquimarinus API 8407116	NR_042375
Enterococcus asini 1_11027	MN822908
Enterococcus avium E16	MK322649
Enterococcus bulliens LMG 28766	NR_145937
Enterococcus caccae 2215-02	NR_043285
Enterococcus camelliae BLN48	MN880432
Enterococcus canintestini 0.3	MK611105
Enterococcus canis NBRC 100695	NR_113931
Enterococcus casseliflavus HWII1-F	LC487888
Enterococcus cecorum A60	NR_024905
Enterococcus columbae DSM 7374	NR_041708
Enterococcus crotali NF45	MT266953
Enterococcus devriesei 790	MT367750
Enterococcus diestrammenae JCM 18359	LC521987
Enterococcus dispar CNM419_12	KC699163
Enterococcus dongliensis 63-4	NR_174223
Enterococcus durans 6895	MT463953
Enterococcus eurekensis 29.3	MN197986
Enterococcus faecalis JCM 5803	NR_040789
Enterococcus faecium IMAU98249	MT473604
Enterococcus florum Gos25-1	LC428281
Enterococcus gallinarum CKY	AB269767
Enterococcus gilvus 3	JN989551
Enterococcus haemoperoxidus NBRC 100709	NR_113936
Enterococcus hawaiiensis AN-16	MN874277
Enterococcus hermanniensis L40	MN904849
Enterococcus hirae MLG3-29-2	MT473367
Enterococcus hulanensis 190-7	NR_174228
Enterococcus innesii RSAA85	OR826331
Enterococcus italicus IMAU98151	MT473506
Enterococcus lactis CK1025	AY683836
Enterococcus lemanii PC32	NR_114648
Enterococcus malodoratus ATCC 43197	NR_114453
Enterococcus massiliensis AM1	NR_144723
Enterococcus mediterraneensis Marseille-P4358	NR_179501
Enterococcus montenegrensis CoE-012-22	OQ627393
Enterococcus moraviensis P37_BA2H	MK883074
Enterococcus mundtii L49	MN904853
Enterococcus nangangensis 94-2	NR_174225
Enterococcus olivae IGG16.11	NR_125610
Enterococcus pallens NBRC 100697	NR_113933
Enterococcus pernyi CS1	FJ555518
Enterococcus phoeniculicola 772	MN513232
Enterococcus pingfangensis 241-2-2	NR_174226

Table S3 (continued). Sequences of the *16S rRNA* gene of *Enterococcus* species used for the design of species-specific primers and TaqMan probes of *E. faecalis* and *E. faecium*.

Species and strains	NCBI accession number
Enterococcus plantarum CCM 7889	NR_118050
Enterococcus pseudoavium EP2-4	MN867717
Enterococcus quebecensis CCRI-16985	NR_117519
Enterococcus raffinosus JCM 8733	LC097071
Enterococcus ratti DS 2705-87	NR_041933
Enterococcus rivorum 756	MT367752
Enterococcus rotai CCM 4630	NR_108137
Enterococcus saccharolyticus JCM 8734	LC097072
Enterococcus saigonensis A28	MT176583
Enterococcus silesiacus R-23712	NR_042405
Enterococcus songbeiensis 85-4	NR_174224
Enterococcus sulfureus NCIMB 13117	NR_119290
Enterococcus termitis LMG 8895	NR_042406
Enterococcus thailandicus S56	JF302672
Enterococcus ureasiticus HYN0074	MF988707
Enterococcus ureilyticus CCM 4629	NR_125485
Enterococcus viikkiensis CNM328_12	KC699114.1
Enterococcus villorum JCM 11557	LC311744.1
Enterococcus wangshanyuanii MN05	NR_159231
Enterococcus xiangfangensis 11097	NR_133741
Enterococcus xinjiangensis JCM 30200	LC590881
