# Supplementary material for: Development of Multiplex RT qPCR Assays for Simultaneous Detection and Quantification of Faecal Indicator Bacteria in Bathing Recreational Waters
Source: Microorganisms. 2024 Jun 18;12(6):1223. doi: 10.3390/microorganisms12061223 (PMC11205496; doi:10.3390/microorganisms12061223)
Supplement: Supplementary file 1 [file microorganisms-12-01223-s001.zip › Table S4.pdf]

Table S4. Sequences of the allantoin transporter-encoding gene (*ybbW*) of *Escherichia coli* strains used for the design of species-specific primers and *E. coli* TaqMan probes.

**Species**	**Strain**	**NCBI accession number**
Escherichia coli	K-12 substr. MG1655	NP_415044
Escherichia coli	K-12 substr. MG1655	AAC73613
Escherichia coli	O26:H11 substr. CVM10026	EIL37346
Escherichia coli	SHECO001	OSK01699
Escherichia coli	ECC-1470	AJG07509
Escherichia coli	BL21	QNG31550
Escherichia coli	O7:K1 str. CE10	AEQ11328
