# Supplementary material for: Development of Multiplex RT qPCR Assays for Simultaneous Detection and Quantification of Faecal Indicator Bacteria in Bathing Recreational Waters
Source: Microorganisms. 2024 Jun 18;12(6):1223. doi: 10.3390/microorganisms12061223 (PMC11205496; doi:10.3390/microorganisms12061223)
Supplement: Supplementary file 1 [file microorganisms-12-01223-s001.zip › Table S5.pdf]

Table S5. Results of the *in silico* amplifications of the *16S rRNA* gene performed with the primers and TaqMan probes of group- and species-specific enterococci, *E. faecalis* and *E. faecium*, using as a template the dataset built with the sequences of 68 *Enterococcus* species accepted by the LPSN - List of Prokaryotic Names with Standing in Nomenclature [39] and carried out in UGENE v33.0 software [44].

**Species**	**Host***	**Source****	**Group- or species-specific**		
			**Enterococci**	***E. faecalis***	***E. faecium***
Enterococcus alcedinis	An (bird)	*Alcedo atthis*	-	-	-
Enterococcus alishanensis	E (vegetal)	VBPs	-	-	-
Enterococcus aquimarinus	E	Sewater	-	-	-
Enterococcus asini	An	Cecium of donkey	-	-	-
Enterococcus avium [a]	H, An	CS, Animal intestines	+	-	-
Enterococcus bulliens	An (mammal)	ABPs from *Camelus* spp	-	-	-
Enterococcus caccae	H, An	Faeces	+	-	-
Enterococcus camelliae	An	ABPs	-	-	-
Enterococcus canintestini	An, E (vegetal)	Faeces, Silage	-	-	-
Enterococcus canis	An	Dog's ear	-	-	-
Enterococcus casseliflavus [a]	H, An, E	Faeces, Water, Plant,	+	-	-
Enterococcus cecorum	An (bird)	Faeces	-	-	-
Enterococcus columbae	An (bird)	Faeces from *Columba* spp	-	-	-
Enterococcus crotali	An (reptile)	Faeces	-	-	-
Enterococcus devriesei [b]	An	Faecal, Bovine, ABPs	+	-	-
Enterococcus diestrammenae	An (insect)	*Diestrammena coreana*	-	-	-
Enterococcus dispar [a]	H, An	CS, ABPs from *Ovis* spp	+	-	-
Enterococcus dongliensis	E (vegetal)	VBPs	-	-	-
Enterococcus durans	H, An	CS, ABPs	-	-	-
Enterococcus eurekensis	An (mammal)	Faeces from *Sus scrofa* spp	-	-	-
Enterococcus faecalis [a]	H, E, An	Faeces, Sewage, ABPs	+	+	-
Enterococcus faecium [a]	H, E, An	Faeces, Sewage, ABPs	-	-	+
Enterococcus florum	E (vegetal)	*Gossypium* spp	-	-	-
Enterococcus gallinarum [a]	H, An	Faeces	+	-	-
Enterococcus gilvus [b]	H, An, E	Faeces, CS, ABPs	+	-	-
Enterococcus haemoperoxidus	E	Water	-	-	-
Enterococcus hawaiiensis	E, An	Water, Faeces	-	-	-
Enterococcus hermanniensis	An	CS	-	-	-
Enterococcus hirae [a]	H, E, An	CS, Water, ABPs and Faeces	-	-	-
Enterococcus hulanensis	E (vegetal)	VBPs	-	-	-
Enterococcus innesii	An (insect)	*Galleria mellonella*	+	-	-
Enterococcus italicus	H, An	CS, ABPs	-	-	-

Information source obtained from Global Catalogue of Microorganisms https://gcm.wdcm.org/

*H: Human; E: Environment; An: Animal;

**CS: Clinical Sample; ABPs: Animal by-products; VBPs: Vegetable by-products.

[a]Pathogenic; [b] Opportunistic pathogen

Table S5 (continued). Results of the *in silico* amplifications of the *16S rRNA* gene performed with the primers and TaqMan probes of group- and species-specific enterococci, *E. faecalis* and *E. faecium*, using as a template the dataset built with the sequences of 68 *Enterococcus* species accepted by the LPSN - List of Prokaryotic Names with Standing in Nomenclature [39] and carried out in UGENE v33.0 software [44].

**Species**	**Host***	**Source****	**Group- or species-specific**		
			**Enterococci**	***E. faecalis***	***E. faecium***
Enterococcus lactis	An	ABPs	-	-	-
Enterococcus larvae	An (insect)	*Allomyrina dichotoma*	-	-	-
Enterococcus lemanii	An (mammal)	Faeces from *Sus scrofa* spp	-	-	-
Enterococcus malodoratus	An	ABPs	-	-	-
Enterococcus massiliensis	H	Faeces	+	-	-
Enterococcus mediterraneensis	H	Faeces	+	-	-
Enterococcus montenegrensis	An	ABPs	-	-	-
Enterococcus moraviensis	E	Water	-	-	-
Enterococcus mundtii	H, An, E	CS, Vegetal, Soil	-	-	-
Enterococcus nangangensis	E (vegetal)	VBPs	-	-	-
Enterococcus olivae	E (vegetal)	*Olea* spp	-	-	-
Enterococcus pallens[b]	H	Faeces, CS	+	-	-
Enterococcus pernyi	An (insecto)	*Antheraea* spp	-	-	-
Enterococcus phoeniculicola	An	CS	-	-	-
Enterococcus pingfangensis	E (vegetal)	VBPs	-	-	-
Enterococcus plantarum	E (vegetal)	*Aegopodium sp.*	-	-	-
Enterococcus pseudoavium [a]	H, An	Faeces, ABPs	+	-	-
Enterococcus quebecensis	E	Well water	-	-	-
Enterococcus raffinosus [a]	H, An	Faeces, CS	+	-	-
Enterococcus ratti	An (mamífero)	Faeces from de *Rattus* spp	-	-	-
Enterococcus rivorum	E	Water	-	-	-
Enterococcus rotai	E	Water, vegetal	-	-	-
Enterococcus saccharolyticus	E	Vegetal, soil	-	-	-
Enterococcus saigonensis [b]	H, An	Faeces, CS, ABPs	+	-	-
Enterococcus silesiacus	E	Inland water, drinking water	-	-	-
Enterococcus songbeiensis	E (vegetal)	VBPs	-	-	-
Enterococcus sulfureus	E	Plant	-	-	-
Enterococcus termitis	An (insecto)	*Isoptera* spp	-	-	-
Enterococcus thailandicus	An	ABPs from *Sus scrofa* spp	-	-	-

Information source obtained from Global Catalogue of Microorganisms https://gcm.wdcm.org/
*H: Human; E: Environment; An: Animal;
**CS: Clinical Sample; ABPs: Animal by-products; VBPs: Vegetable by-products.
[a]Pathogenic; [b] Opportunistic pathogen

Table S5 (continued). Results of the *in silico* amplifications of the *16S rRNA* gene performed with the primers and TaqMan probes of group- and species-specific enterococci, *E. faecalis* and *E. faecium*, using as a template the dataset built with the sequences of 68 *Enterococcus* species accepted by the LPSN - List of Prokaryotic Names with Standing in Nomenclature [39] and carried out in UGENE v33.0 software [44].

**Species**	**Host***	**Source****	**Group- or species-specific primers and TaqMan probes**		
			Enterococci	***E. faecalis***	***E. faecium***
Enterococcus ureasiticus	E	Well water	-	-	-
Enterococcus ureilyticus	E	Inland Water, Drinking water, Vegetal	-	-	-
Enterococcus viikkiensis [b]	An	ABPs	+	-	-
Enterococcus villorum	An (mamífero)	Faeces from *Sus scrofa* spp	-	-	-
Enterococcus wangshanyuanii	An (mamífero)	Faeces from *Bos* spp	-	-	-
Enterococcus xiangfangensis	E	VBPs	-	-	-
Enterococcus xinjiangensis	An	ABPs	-	-	-

Information source obtained from Global Catalogue of Microorganisms https://gcm.wdcm.org/

*H: Human; E: Environment; An: Animal;

**CS: Clinical Sample; ABPs: Animal by-products; VBPs: Vegetable by-products.

[a]Pathogenic; [b] Opportunistic pathogen
