# Supplementary material for: Development of Multiplex RT qPCR Assays for Simultaneous Detection and Quantification of Faecal Indicator Bacteria in Bathing Recreational Waters
Source: Microorganisms. 2024 Jun 18;12(6):1223. doi: 10.3390/microorganisms12061223 (PMC11205496; doi:10.3390/microorganisms12061223)
Supplement: Supplementary file 1 [file microorganisms-12-01223-s001.zip › Table S6.pdf]

Table S6. Results of *in silico* amplification of the allantoin transporter-encoding gene (*ybbW*) for species-specific *E. coli* assay performed in UGENE v33.0 sotware [44] and the In Silico PCR Amplification tool [45].

**Species and strain**	**Species-specific primers and TaqMan probes of *E. coli***
Escherichia blattae DSM 4481	-
Escherichia coli AE1-2	+
Escherichia coli O157:H7	+
Escherichia coli XJ133-127-1NF1	+
Escherichia coli K-12 / MG1655	+
Escherichia coli K-12 / W3110	+
Escherichia coli 0127:H6 E2348/69	+
Escherichia coli 042	-
Escherichia coli 536	+
Escherichia coli 55989	+
Escherichia coli ABU 83972	+
Escherichia coli APEC O1	+
Escherichia coli APEC O78	+
Escherichia coli ATCC 8739	+
Escherichia coli ATCC 11775	+
Escherichia coli BREL606	+
Escherichia coli BL21(DE3)	+
Escherichia coli BL21-Gold(DE3)pLysS AG	+
Escherichia coli BW2952	+
Escherichia coli CFT073	+
Escherichia coli DH1	+
Escherichia coli E24377A	+
Escherichia coli ED1a	+
Escherichia coli ETEC H10407	+
Escherichia coli HS	+
Escherichia coli IAI1	+
Escherichia coli IAI39	-
Escherichia coli IHE3034	+
Escherichia coli JJ1886	+
Escherichia coli K-12 / *W3110*	+
Escherichia coli KO11FL	+
Escherichia coli LF82	+
Escherichia coli LY180	+
Escherichia coli NA114	+
Escherichia coli O103:H2 12009	+
Escherichia coli O104:H4 2009EL-2050	+
Escherichia coli O104:H4 2009EL-2071	+
Escherichia coli O104:H4 2011C-3493	+
Escherichia coli O111:H- 11128	+
Escherichia coli O157:H7 EDL933	+
Escherichia coli O157:H7 EC4115	+
Escherichia coli O157:H7 Sakai	+

Table S6 (continued). Results of *in silico* amplification of the allantoin transporter-encoding gene (*ybbW*) for species-specific *E. coli* assay performed in UGENE v33.0 sotware [44] and the In Silico PCR Amplification tool [45].

**Species and strain**	**Species-specific primers and TaqMan probes of *E. coli***
Escherichia coli O157:H7 TW14359	+
Escherichia coli O26:H11 11368	+
Escherichia coli O157:H7 TW14359	+
Escherichia coli O26:H11 11368	+
Escherichia coli O55:H7 CB9615	+
Escherichia coli O55:H7 RM12579	+
Escherichia coli O7:K1 CE10	-
Escherichia coli O83:H1 NRG 857C	+
Escherichia coli P12b	+
Escherichia coli PMV-1	+
Escherichia coli S88	+
Escherichia coli SE11	+
Escherichia coli SE15	+
Escherichia coli SMS-3-5	+
Escherichia coli UM146	+
Escherichia coli UMN026	+
Escherichia coli UMNK88	+
Escherichia coli UTI89	+
Escherichia coli W	+
Escherichia coli Xuzhou21	+
Escherichia coli K-12 / DH10B	+
Escherichia coli K-12 / MDS42 DNA	+
Escherichia coli D i14	+
Escherichia coli D i2	+
Escherichia fergusonii 190311L245	-
Escherichia fergusonii ATCC 35469	-
Escherichia marmotae HT073016	-
Escherichia marmotae NCTC8196	-
Shigella boydii P288	-
Shigella boydii CDC 3083-94	-
Shigella boydii Sb227	-
Shigella dysenteriae FBD015	-
Shigella dysenteriae 1617	-
Shigella dysenteriae Sd197	-
Shigella flexneri GH24	-
Shigella flexneri 2002017	-
Shigella flexneri 2a 301	-
Shigella flexneri 2a 2457T	-
Shigella flexneri 5 8401	-
Shigella sonnei CECT 4887	-
Shigella sonnei 53G	-
Shigella sonnei Ss046	-
