# Supplementary figures and images for: Development of Multiplex RT qPCR Assays for Simultaneous Detection and Quantification of Faecal Indicator Bacteria in Bathing Recreational Waters
Source: Microorganisms. 2024 Jun 18;12(6):1223. doi: 10.3390/microorganisms12061223 (PMC11205496; doi:10.3390/microorganisms12061223)

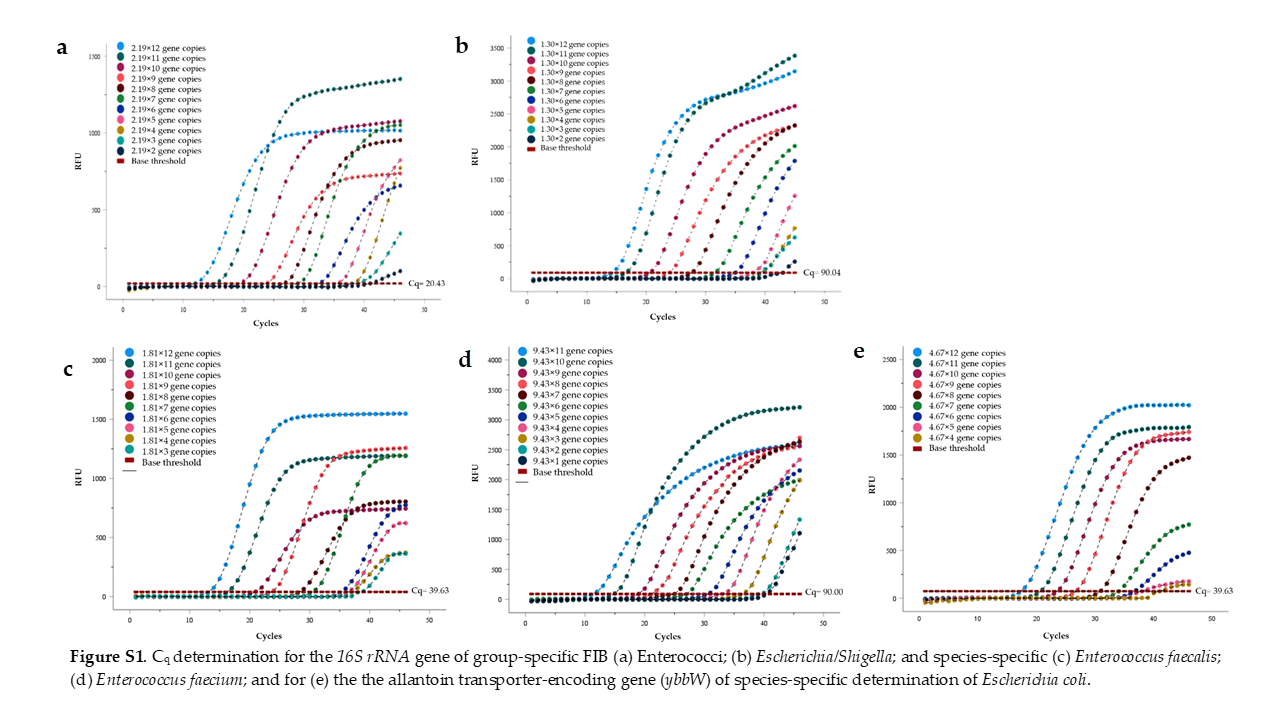

Supplement: Supplementary file 1 [file microorganisms-12-01223-s001.zip › Figure S1.tif]
